# Systemic Stimulation of TLR2 Impairs Neonatal Mouse Brain Development

**DOI:** 10.1371/journal.pone.0019583

**Published:** 2011-05-06

**Authors:** Xiaonan Du, Bobbi Fleiss, Hongfu Li, Barbara D'angelo, Yanyan Sun, Changlian Zhu, Henrik Hagberg, Ofer Levy, Carina Mallard, Xiaoyang Wang

**Affiliations:** 1 Perinatal Center, Department of Neuroscience and Physiology, University of Gothenburg, Gothenburg, Sweden; 2 Department of Pediatrics, the Third Affiliated Hospital of Zhengzhou University, Zhengzhou, People's Republic of China; 3 Center for Brain Repair and Rehabilitation, Institute of Neuroscience and Physiology, University of Gothenburg, Gothenburg, Sweden; 4 Perinatal Center, Department of Obstetrics and Gynecology, University of Gothenburg, Gothenburg, Sweden; 5 Institute of Reproductive and Developmental Biology, Imperial College, London, United Kingdom; 6 Department of Medicine, Division of Infectious Diseases, Children's Hospital Boston and Harvard Medical School, Boston, Massachusetts, United States of America; Columbia University, United States of America

## Abstract

**Background:**

Inflammation is associated with perinatal brain injury but the underlying mechanisms are not completely characterized. Stimulation of Toll-like receptors (TLRs) through specific agonists induces inflammatory responses that trigger both innate and adaptive immune responses. The impact of engagement of TLR2 signaling pathways on the neonatal brain is still unclear. The aim of this study was to investigate the potential effect of a TLR2 agonist on neonatal brain development.

**Methodology/Principal Findings:**

Mice were injected intraperitoneally (i.p.) once a day from postnatal day (PND) 3 to PND11 with endotoxin-free saline, a TLR2 agonist Pam_3_CSK_4_ (5 mg/kg) or Lipopolysaccharide (LPS, 0.3 mg/kg). Pups were sacrificed at PND12 or PND53 and brain, spleen and liver were collected and weighed. Brain sections were stained for brain injury markers. Long-term effects on memory function were assessed using the Trace Fear Conditioning test at PND50. After 9 days of Pam_3_CSK_4_ administration, we found a decreased volume of cerebral gray matter, white matter in the forebrain and cerebellar molecular layer that was accompanied by an increase in spleen and liver weight at PND12. Such effects were not observed in Pam3CSK4-treated TLR 2-deficient mice. Pam3CSK4-treated mice also displayed decreased hippocampus neuronal density, and increased cerebral microglia density, while there was no effect on caspase-3 or general cell proliferation at PND12. Significantly elevated levels of IL-1β, IL-6, KC, and MCP-1 were detected after the first Pam3CSK4 injection in brain homogenates of PND3 mice. Pam_3_CSK_4_ administration did not affect long-term memory function nor the volume of gray or white matter.

**Conclusions/Significance:**

Repeated systemic exposure to the TLR2 agonist Pam_3_CSK_4_ can have a short-term negative impact on the neonatal mouse brain.

## Introduction

Improved neonatal and intensive care has enabled the survival of preterm infants with very low birth weights. These infants are at increased risk for nosocomial infection, and *Staphylococcus* epidermidis is the predominant pathogen isolated from blood cultures obtained in the neonatal intensive care unit [1], [2], [3], [4], [5]. Increasing evidence suggests that neonatal brain injury is associated with infection/inflammation, but the underlying mechanisms are incompletely characterized [6], [7], [8]. Preterm infants in particular have an increased risk of brain injury, which is predominantly located in the cerebral white matter, although recently a high frequency of grey matter injury has also been reported [9]. Moreover, very low birth weight premature infants manifest cerebellar abnormalities [6].

Infection/inflammation stimulates innate and subsequent adaptive immune responses via the Toll-like Receptor (TLR) family of pattern-recognition receptors that can be stimulated with specific agonists. TLRs exist in a wide range of tissues outside the immune system, including the central nervous system (CNS). TLR2 forms heterodimers with TLR1 and TLR6, and these receptor complexes recognize molecules expressed on Gram-positive bacteria, such as peptidoglycan, lipopeptides, and lipoproteins, and they also mediate recognition of whole bacteria such as *Staphylococcus epidermidis* [10], [11], [12]. Of note, TLR2 is selectively up-regulated in the peripheral blood mononuclear cells of human newborns infected with Gram-positive bacteria [13]. With respect to the CNS, a role for TLR2 signaling in adult mouse brain injury has been suggested, as summarized by a most recent review [14] but there are few reports that define the role of TLR2 signaling in neonatal brain injury. However, there are studies that suggest that TLR2 and TLR4 are the principal TLRs present on microglia which are involved in the innate immune response to infection/hypoxia-ischemia; for a most recent review, please see [15]. Of note, neonates demonstrate a distinct functional expression of the TLR system [16], [17], and therefore studies of outcome in adult models cannot be directly extrapolated to newborns. In the present study, we hypothesized that stimulation of TLR2 during a critical period of neonatal brain development would have a detrimental effect on the immature brain, which may be measurable as changes in adult behavior. We used a synthetic lipopeptide, Pam_3_CysSerLys_4_ (Pam_3_CSK_4_), as a specific TLR2 agonist [18], that was administrated systemically to newborn wild-type and TLR2 deficient mice from postnatal day (PND) 3 to PND11 to evaluate short and long-term effects on the developing mouse brain.

## Methods

### Ethics statement

The animal experiments were approved by the local Animal Ethics Committee at the University of Gothenburg (Ethical approval 350-2009).

### Animals

Time-mated pregnant C57BL/6 wild-type mice were purchased from Charles River Laboratories (Sulzfeld, Germany) and gave birth in the animal facility (Experimental Biomedicine, University of Gothenburg, Gothenburg, Sweden). B6.129-Tlr2*^tm1Kir^*/J (TLR2 –deficient) mice were purchased from the Jackson Laboratory (US) and bred in the animal facility. The day of birth was defined as postnatal day (PND) 0. Mice were housed with a 12-hour light/dark cycle with ad libidium access to a standard laboratory chow diet (B&K, Solna, Sweden) and drinking water was provided.

### Drug administration

Offspring of both genders of C57BL/6 wild type mice were randomly divided into three groups. i) Negative control mice treated with endotoxin-free saline (10 µl/g, sb776, Sigma, USA, n = 12); ii) Pam_3_CSK_4_ (5 mg/kg, Invitrogen, n = 11) treated mice; and iii) Lipopolysaccharide, (LPS, 0.3 mg/kg, Escherichia coli 055:B5; Sigma, Stockholm, Sweden) injected mice (n = 13). LPS animals were used for comparison, as we have previously shown that repeated administration of this dose of LPS from PND3 to PND11 induces neonatal brain white/gray matter injury [19]. Offspring of both genders of TLR2-deficient mice were randomly divided into two treatment groups: i) Pam_3_CSK_4_, treated (5 mg/kg, n = 8) mice; ii) endotoxin-free saline treated (10 µl/g, sb776, Sigma, USA, n = 10). Mice were injected intraperitoneally (i.p.) once a day from PND3 to PND11. Pups were sacrificed at PND12 and PND53 and brain (including cerebrum and cerebellum), spleen, and liver were collected and weighed.

### Immunohistochemical staining

Mice at PND12 and PND53 were deeply anesthetized and perfused intracardially with saline followed by 5% buffered formaldehyde (Histofix; Histolab, Gothenburg, Sweden). Brains were removed and fixed in 5% buffered formaldehyde for 18–24 hours and processed to paraffin. The cerebrum was cut into 10-µm coronal sections and collected at 50-section intervals. Serial sections were used for histologic stains, as previously described [20]. Briefly, nonspecific binding was blocked for 30 minutes with 4% horse serum or 4% goat serum in phosphate-buffered saline. The following primary antibodies were used: microtubule associated protein-2 (MAP-2; clone HM-2, Sigma), rabbit anti-myelin basic protein (MBP, Sternberger Monoclonal Incorporated, SMI 94, Lutherville, Massachusetts), active form of caspase-3 (557038, BD Bioscience Pharmingen); anti-neuronal nuclear antigen (NeuN) (MAB377B, Chemicon), anti-Ki67 (NCL-KI-67-MMI, Novocasta), and anti-Iba-1 (019-19741 Wako). Primary antibodies were incubated for 60 minutes at room temperature followed by the corresponding biotinylated secondary antibodies (all from Vector, Burlingame, California) also for 60 minutes at room temperature. Visualization was performed using Vectastain ABC Elite with 0.5 mg/mL 30-diaminobenzidine enhanced with 15 mg/mL ammonium nickel sulfate, 2 mg/mL β-D-glucose, 0.4 mg/mL ammonium chloride, and 0.01 mg/mL β-glucose oxidase (all from Sigma).

The cerebellum from PND12 mice was cut into 10 µm sagittal sections and collected at 50-section intervals. Serial sections were used for thionin/fuchsin acid staining as described previously [21].

### Gray and white matter volume measurement

The forebrain gray matter area was determined by measuring the MAP-2 immunoreactive area from 6 serial sections per animal. The cerebral subcortical white matter area was determined by measuring MBP immunoreactive area in 6 serial sections per animal. The area of the molecular cell layer and granule cell layer of the cerebellum were measured in thionin/fuchsin acid stained sections in 8 serial sections. Micro Image, version 4.0 (Micro-Macro AB, Gothenburg, Sweden) was used for all the above measurements. The volume was calculated from area measurements according to the Cavalieri's Principal as described previously [20], using the following formula: V = SA · p · T, where V is the total volume, SA is the sum of the areas measured, p is the inverse of the section sampling fraction, and T is the section thickness.

### Cell counting

NeuN-positive cells were counted in all *cornu ammonis* (CA) fields and dentate gyrus (DG) in 2 sections through the anterior hippocampus and Iba-1 positive cells in the right hemisphere in 4 sections, using stereological principles (Stereo investigator 7, System Inc, Magdeburg, Germany), with a counting frame of 40×40 µm per section for NeuN, and 150×150 µm per section for Iba-1. Ki67-positive cells were counted within the area of the granule cell layer (GCL), including the subgranular zone (SGZ) in the DG and CA of the hippocampus. Caspase-3 positive cells were counted in the right hemisphere (4 levels) and subcortical white matter (4 levels) and Purkinje cells in the posterior lobes in all 8 serial sections of the cerebellum. The average number of positive cells/mm^2^ was calculated.

### Cytokine/chemokine assay

Cytokine/chemokines were measured in whole brain homogenate supernatants from PND3 wild type mice sacrificed 6 hours after i.p. treatment with endotoxin-free saline (10 µl/g, n = 6), LPS (0.3 mg/kg; n = 7) or Pam_3_CSK_4_ (5 mg/kg; n = 7). Mice were deeply anesthetized and perfused intracardially with saline and brains were removed and snap frozen. Brains were homogenized by sonication in ice-cold homogenization buffer containing 1% protease inhibitor cocktail (P8340, Sigma-Aldrich) and 3% EDTA in 0.1 M phosphate buffered saline, centrifuged at 4°C once at 900×*g* for 10 minutes and then at 10,000×*g* for 15 minutes, supernatants were collected and stored at −80°C until use. Concentrations of IL-1β, IL-6, KC, MCP1, IL-10, IL-17 and TNFα were measured using Bio-plex Multiplex Cytokine Assay (Bio-Rad laboratories, Hercules, CA). Results were normalized to the amount of protein per well, as determined using a Bio-Rad DC protein assay.

### Trace Fear Conditioning test

Long term memory function was measured via Trace Fear Conditioning test at PND50, in an Automatic Reflex Conditioner 7531 (inside dimensions 390×95×165 mm; Cat No: 7530, Ugo Basile, Italy) as previously described [22], [23] with some modifications. Animals were timed for freezing within a 2 min time period recorded by digital video cameras. Freezing was defined as absence of movement except for respiration. The procedure was conducted over 2 days. On day 1 freezing was scored prior to mice receiving a pairing of a tone (20 seconds, 80 dB, 670 kHz) and a shock (2 seconds, 0.5 mA). The time interval between the tone and the shock was 2 seconds. On day 2, freezing was scored pre-tone and the tone was then presented once for 30 seconds, 80 dB, 670 kHz. No shock was administered and freezing was scored for 2 minutes after the tone presentation (tone-elicited freezing, post-tone freezing).

### Statistics

Statistical Package for the Social Sciences (*SPSS* 17.0) and *StatView* (5.0.1) were used for all analyses. One-way ANOVA followed by LSD post hoc test was used for comparison of data from more than two groups. For all other analysis, Student's unpaired t-test was used for comparison. Results are presented as mean ± standard error of the mean (SEM). P<0.05 was considered statistically significant.

## Results

### Brain, liver, and spleen weight changes at PND12

**Figure 1:**
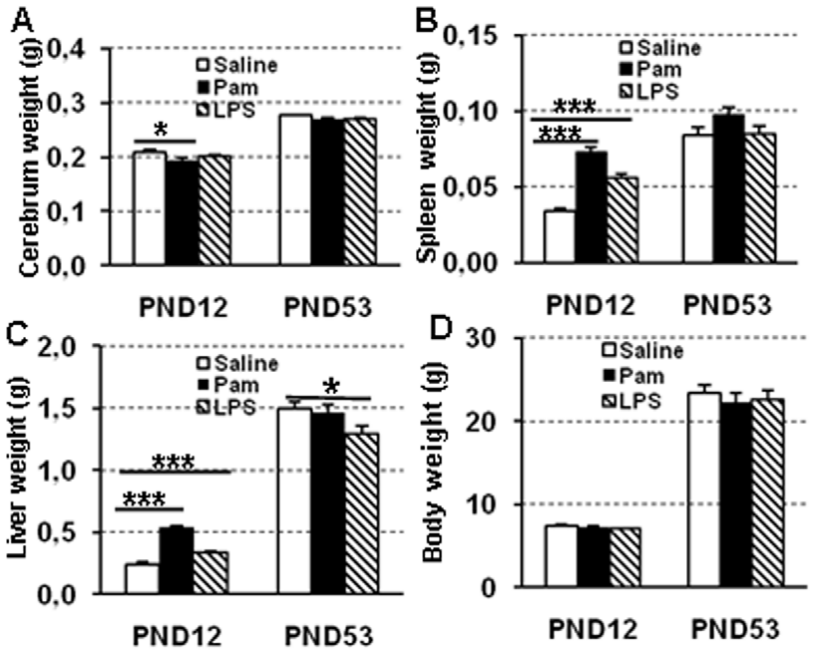
Pam3CSK4 alters brain, spleen and liver weights in neonatal mice. Quantitative analysis of the cerebral weight (A), spleen weight (B), liver weight (C), body weight (D) at PND12 and PND53 after Pam3CSK4 administration from PND 3 to 11 in while type mice. *p<0.05; ***p<0.001.

After repeated administration of 5 mg/kg Pam3CSK4 once a day from PND3 to PND11, brain weight was decreased compared with endotoxin-free saline-treated animals at PND12. In contrast, there was no difference between endotoxin-free saline-treated animals and LPS-treated animals (Figure 1A). We found no infarctions, dilatation of the cerebral ventricles, or morphological signs of cell death in any of the brain regions examined after administration of Pam_3_CSK_4_ or LPS.

There was a significant increase in both the absolute spleen and liver weights in animals treated with Pam_3_CSK_4_ and LPS compared with those treated with endotoxin-free saline at PND12 (Fig. 1B, 1C) as well as the relative spleen and liver weight to body weight ratio (data not shown). The whole body weight was not different between groups at PND12 (Fig. 1D). No mortality or other signs of morbidity were found during the entire study period.

### Gray and white matter changes in the cerebrum at PND12

**Figure 2:**
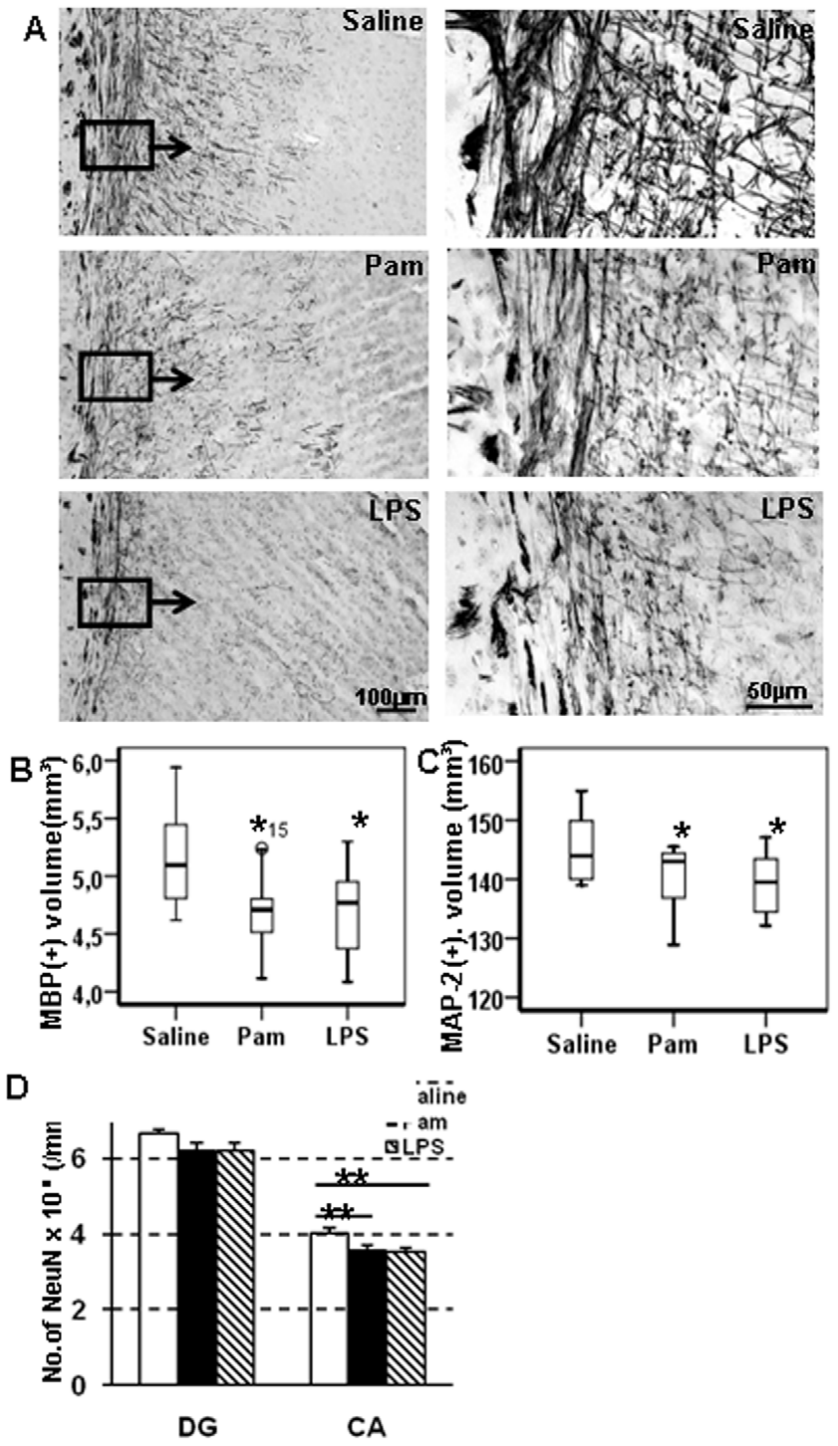
Pam3CSK4 alters brain development. Representative microphotographs of MBP staining in the subcortical area (A), Quantitative analysis of subcortical white matter volume (B) cerebral gray matter volume (C) and the number of NeuN positive cells in both DG and CA of hippocampus(D), at PND 12 after Pam_3_CSK_4_ administration from PND3 to PND11 in while type mice. *p<0.05; ** p<0.01.

**Figure 3:**
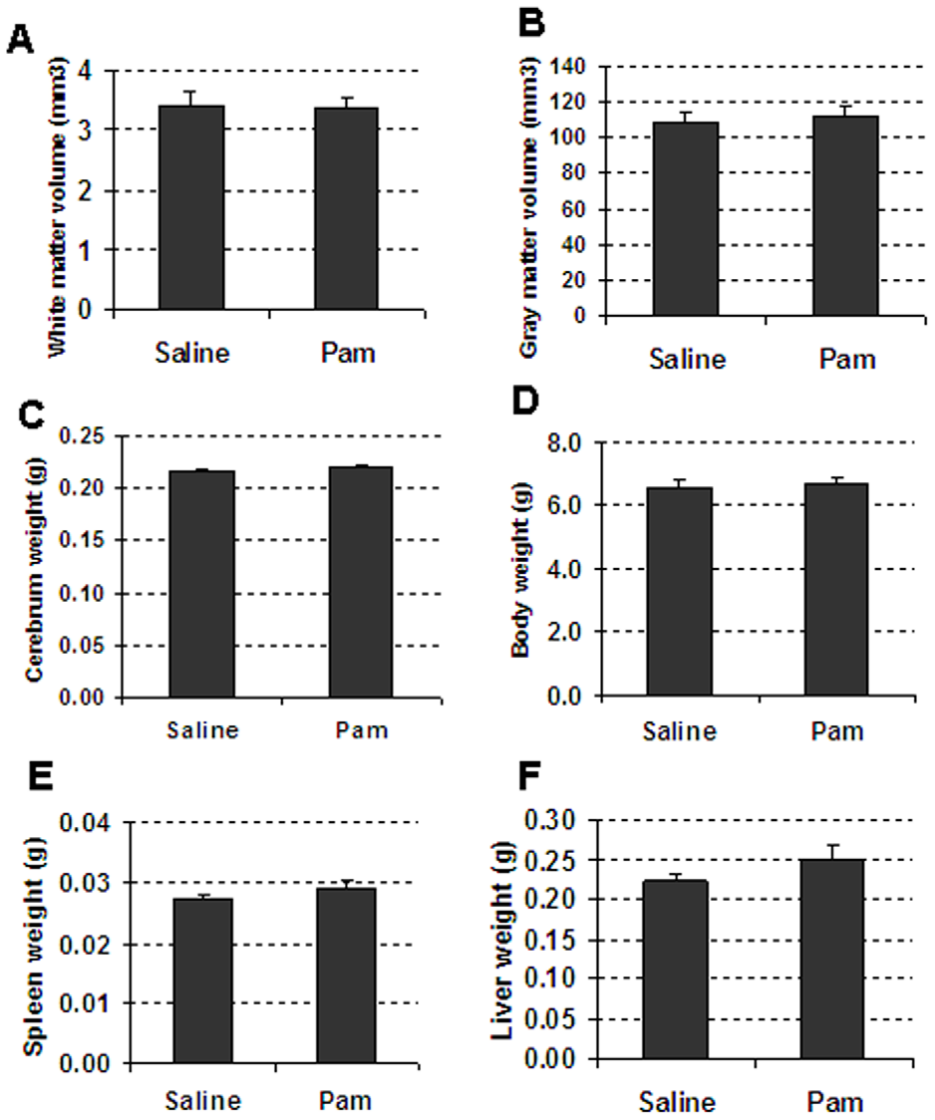
Pam3CSK4 does not affect brain development in TLR2 deficient mice. Quantitative analysis of subcortical white matter volume (A) cerebral gray matter volume (B) cerebral weight (C), body weight (D) spleen weight (E), and liver weight (F) at PND12 after Pam3CSK4 administration from PND 3 to 11 in TLR2 deficient mice.

To examine the gray matter and white matter changes after Pam_3_CSK_4_ treatment, the cerebral gray matter volume was measured using immunohistochemical staining for the neuronal marker MAP-2, and subcortical white matter volume was measured using the myelin marker MBP (Fig. 2A). At PND12, significantly decreased cerebral white matter (Fig. 2B) and gray matter volume (Fig. 2C) were found both in the Pam_3_CSK_4_ and LPS-treated mice compared with saline-treated mice. In contrast, Pam_3_CSK_4_ administration to TLR 2 -deficient mice from PND3 to PND11 once a day did not result in any significant differences between the Pam_3_CSK_4_ treatment group and saline controls, with respect to both the white (Fig. 3A) and gray (Fig. 3B) matter volume. Similarly, brain weight (Fig. 3C), body weight (Fig. 3D), spleen weight (Fig. 3E) and liver weight (Fig. 3F) also did not change in the Pam_3_CSK_4_-injected TLR2-deficient animals compared to endotoxin-free saline-treated animals. These findings further confirm that the observed white/gray matter changes following Pam_3_CSK_4_ administration in wild type mice are TLR2- dependent.

**Figure 4:**
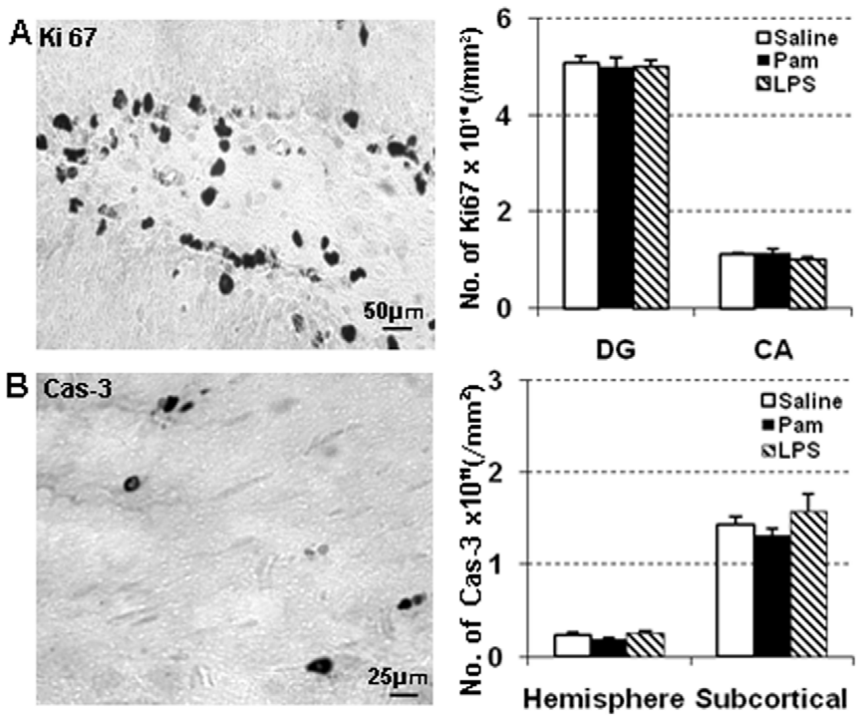
Pam3CSK4 has no effect on proliferation or apoptosis. Representative photomicrographs and quantitative analysis of Ki67 (A), and active Caspase-3(B) at PND 12 after Pam_3_CSK_4_ administration from PND 3 to PND11 in while type mice. ** p<0.01. The pictures show representative positive staining of Ki67 (A) and caspase-3 (B).

LPS-induced inflammation reduces hippocampal neurogenesis in adult rats [24]. To investigate the specific impact of Pam_3_CSK_4_-exposure on post-mitotic neurons in the hippocampus, NeuN positive cells were counted in the dentate gyrus (DG) and the CA fields. The density of NeuN-positive cells in the CA fields was significantly decreased both in Pam_3_CSK_4_ and LPS-treated mice compared with endotoxin-free saline-treated animals at PND12 (Fig. 2D). There was no difference in the density of NeuN positive cells in the DG between groups (Fig. 2D). Cell proliferation and apoptosis at PND12 were examined by staining brain sections with cell proliferation marker Ki67, and the apoptosis marker active caspase-3. There were no significant differences in number of Ki67 positive cells (Fig. 4A) or number of active caspase-3 positive cells in either the cerebral gray matter or the subcortical white matter among the three groups (Fig. 4B).

### Inflammation after Pam3CSK4 administration

**Figure 5:**
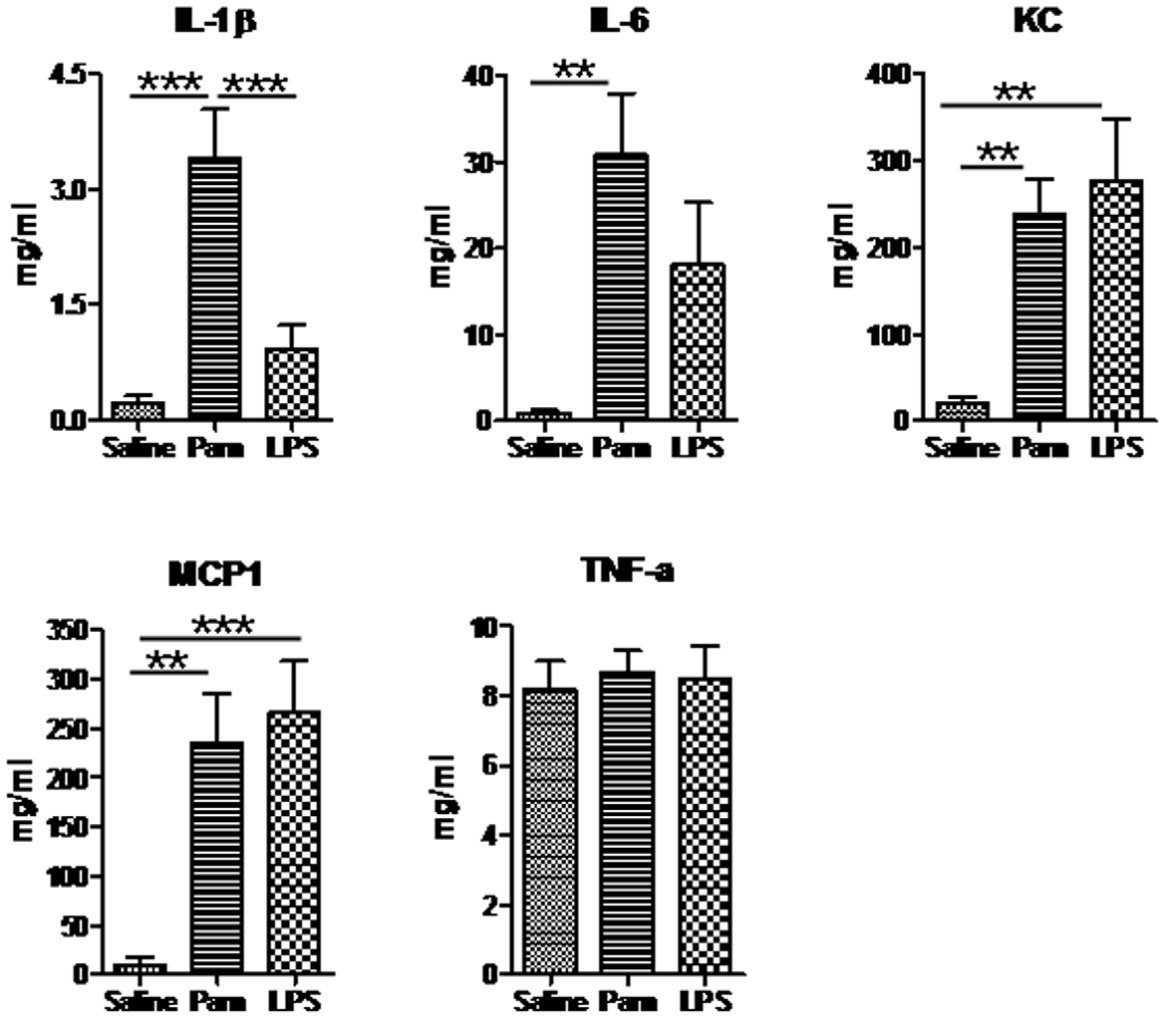
Pam3CSK4 induces brain cytokine production. Cytokine/chemokine changes in brain homogenates at 6 hours after the first Pam3CSK4 administration at PND3 in while type mice. *, p<0.05; **, p<0.01; ***, p<0.001.

To characterize the inflammatory response after Pam_3_CSK_4_ treatment, we first analyzed the cytokine/chemokine production by multiplex ELISA in brain homogenate samples at 6 hours after the first Pam_3_CSK_4_ treatment at PND3, in comparison with saline and LPS treated mice. It was found that 5 mg/kg Pam3CSK4 treatment induced elevated levels of IL-1ß, IL-6, KC, MCP-1, similar to those cytokines and chemokines induced by 0.3 mg/kg LPS (Fig. 5). IL-1ß was an exception in that a significant increase was noted in Pam_3_CSK_4_-treated pups compared with LPS-treated pups. Of note, IL-6 was significantly increased by Pam_3_CSK_4_ but not by LPS. TNF-α levels did not change in either of the two treatment groups. IL-10 and IL-17 levels were below the limits of detection in all brain homogenate samples tested.

**Figure 6:**
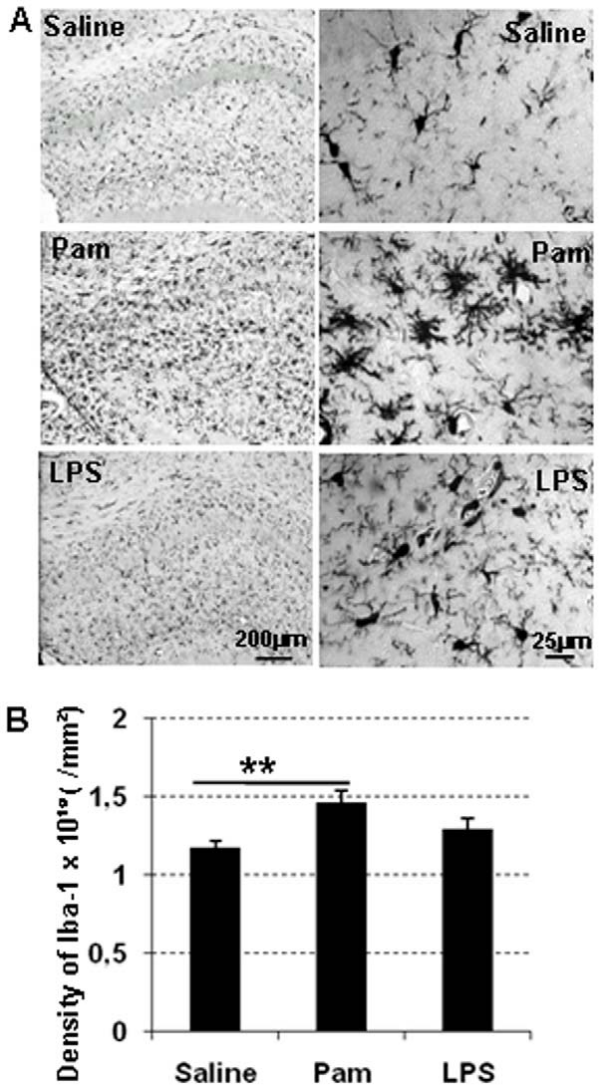
Pam3CSK4 induces microglial activation. Representative microphotograph of Iba-1 in the hippocampus area (A) and Iba-1 positive cells counts (B) at PND 12 after Pam_3_CSK_4_ administration from PND 3 to PND11 in while type mice. **p<0.01.

To further examine the inflammatory response, we stained brain sections for the microglia marker Iba-1 (Fig. 6A). There was a significant increase of Iba-1 positive cells in the Pam_3_CSK_4_-treated group compared with endotoxin-free saline treated animals, while there was no difference between the LPS-treated group and endotoxin-free saline group (Fig. 6B).

### Cerebellar changes at PND12

**Figure 7:**
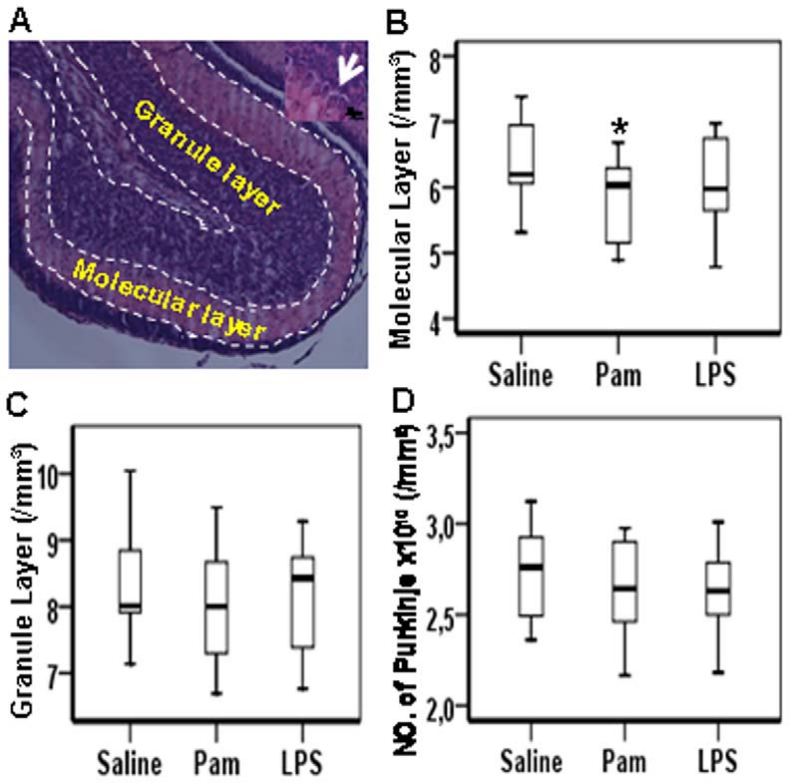
Pam3CSK4 decreases cerebellar molecular layer volume. Representative microphotograph of the cerebellar lobe (A) with arrow indicating Purkinje cell under higher magnification. Quantitative analysis of the total volume of molecular cell layer (B), granule cell layer (C), and Purkinje cell counts (D) in cerebellum at PND 12 after Pam_3_CSK_4_ administration from PND 3 to PND 11 in while type mice. Arrow indicates Purkinje cell with higher magnification. *p<0.05.

To investigate the effect of Pam_3_CSK_4_ administration on the neonatal cerebellum, molecular cell layer and granule cell layer volumes were measured and the density of Purkinje cells was counted (Fig. 7A). There was a significant decrease in the molecular cell layer volume in Pam_3_CSK_4_-treated mice but not LPS treated mice compared with saline-treated pups (Fig. 7B), while there were no differences in the granule cell layer volume (Fig. 7C) or the number of Purkinje cells between the three groups (Fig. 7D).

### Long-term effects of neonatal Pam3CSK4 administration

**Figure 8:**
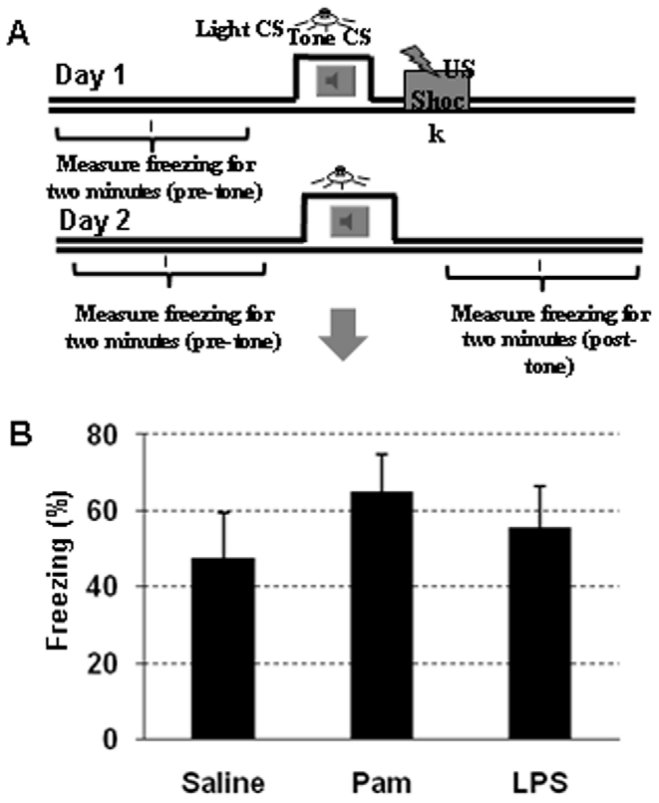
Pam3CSK4 has no effect on adult hippocampal dependent learning. Schematic diagram of Trace Fear Conditioning test (A), US: unconditioned stimulus, the foot shock. CS: conditioned stimulus, a particular context and/or a cue. Histograms displays post-tone freezing in the Trace Fear Conditioning test at PND50 (B) in while type mice.

Since we found a decrease in gray and white matter volumes and a decrease in the number of neurons in hippocampus at PND 12 after Pam_3_CSK_4_ administration, we examined whether these early brain alterations persisted to young adulthood and related to hippocampus-dependent learning and memory deficits. To examine any long term effect of Pam_3_CSK_4_ administration and associated neonatal brain injury on learning and memory function, the Trace Fear Conditioning test was conducted at PND50 (Fig. 8A). At PND53, mice were killed and the weight of the cerebrum, liver, spleen and the whole body were measured. In addition, signs of brain injury for both gray and white matter injury were examined.

For the Trace Fear Conditioning test, no significant differences were found between the three groups (Fig. 8B). At PND53, the weight of the cerebrum, liver, spleen, and body were not different between groups, except for a decreased liver weight in the LPS-treated group (P = 0.025, Fig. 1C). Neither gray matter nor white matter volumes were different among all three groups (data not shown).

## Discussion

In the present study, we found that repeated systemic administration of a TLR2 agonist induced elevated cytokine/chemokine levels in brain homogenates, reduced neonatal gray and white matter volume and hippocampal neuron density, and increased number of microglia cells. By adulthood, brain injury had recovered and there was no detectable long-term change in memory function. To our knowledge, this is the first report of the role of TLR2 agonists on short and long term neonatal brain development. The present study provides important direct evidence that systemic inflammation via TLR2 may exert negative effects on neonatal brain development.

In the rodent, there is a major growth spurt of the brain in the first postnatal week [25], which equates to the second-third trimester in human pregnancy, a developmental window when white matter damage or deficiency of white matter growth is presumed to occur in the human. We used a repeated Pam_3_CSK_4_ exposure model from PND3 up to PND11, therefore, covering the period of rapid brain growth in rodents.

To ensure a biologic effect, we used a relatively high dose of Pam_3_CSK_4_ (5 mg/kg) compared with other in vivo studies in the adult, that range from 5 µg/kg to 2 mg/kg [26], [27], [28], [29]. However, 5 mg/kg Pam_3_CSK_4_ and 0.3 mg/kg LPS treatment produced almost identical levels of KC and MCP-1 in brain homogenates, and despite this relatively high dose, we found no mortality or other signs of morbidity. Similarly, in previous studies we found no adverse effects using the same dose of the TLR2 agonist Lipoteichoic acid (LTA) [30]. These observations suggest that TLR2 agonists have relatively lower potency in neonatal mice compared with the TLR4 agonist LPS.

TLR2 mRNA and protein is expressed in the cortex in embryonic and early postnatal stages of development [31], with relatively low expression before birth that increases during the first 2 weeks of life [32]. Loss of TLR2 does not appear to result in direct defects in cerebral development [31]. However, TLR2 mRNA is expressed constitutively in the adult mouse brain [33] and TLR2 deficiency results in impaired neurogenesis in the hippocampus by adulthood [34]. TLR2 mRNA and genes related to the TLR2 signaling pathway was shown to be induced in the ipsilateral mouse brain hemispheres after transient middle cerebral artery occlusion (MCAO) [35], [36]. Moreover, adult TLR2-deficient mice demonstrated reduced brain damage and improved functional outcome after MCAO [35], [36], [37], though contradictory results have demonstrated a TLR-2 dependent increased brain infarct size after cerebral ischemia/reperfusion injury [38]. Further, in adult mice, hyaluronan blocks oligodendrocyte progenitor maturation and remyelination through TLR2 pathway [39]; Intrathecal administration of Pam_3_CSK_4_ induces the pathophysiological hallmarks of bacterial meningitis and neuronal damage in a TLR2-dependent fashion in adult rats [40]. Overall, these observations suggest that activation of TLR2 may be detrimental in acute CNS injury, and together with our observations that a TLR2 agonist causes neonatal brain injury, is suggestive that TLR2 antagonists may have potential as novel neuroprotective agents.

Pam_3_CSK_4_ is a synthetic tripalmitoylated lipopeptide that mimics the acylated amino terminus of bacterial lipoproteins, such as is found on the nosocomial pathogen *Staphylococcus* epidermidis. Pam_3_CSK_4_ is specifically recognized by a heterodimer of TLR2 and TLR1, stimulation then resulting in the activation of intracellular signaling events. Although there are few studies on the role of TLR2 in immature brain injury, the TLR2 pathway is thought to play a role in Group B streptococcus-induced neurodegeneration [41]. Moreover, in postnatal day 11 rats, intracisternal injection with 0.5 µg of Pam_3_CSK_4_ in the infant rat model of experimental pneumococcal meningitis is capable of inducing a neuroinflammatory response but does not induce hippocampal apoptosis [42]. However, the TLR2 agonist LTA, does not affect vulnerability to hypoxia-ischemia in immature rats [30] and deletion of the gene encoding the adaptor protein MyD88, important for signaling downstream of TLR2 and other TLRs, did not protect the immature brain from hypoxic-ischemic brain injury [14]. Together with our present findings, showing that forebrain and cerebellar volume were recovered by PND53, this suggests that the role of TLR2 in brain injury may be context dependent, with a role in neurodegeneration by whole bacteria that may require engagement of multiple pattern recognition receptors, including several TLRs, NOD-like receptors (NLRs), and complement systems, but a more limited, reversible effect in the context of a pure TLR2 agonist. Similarly, following prenatal stimulation of TLR4 by LPS, there is a transient decrease in myelination and functional outcome which is reversed later in development [43].

Demonstrating the transient effects of Pam_3_CSK_4_ on brain injury, the Trace Fear Conditioning test did not detect any learning and memory deficit in young adulthood. Trace Fear Conditioning to either a cue or a context represents a form of associative learning and memory test that has been well characterized in many species [44], and used as a sensitive method to detect hippocampus-dependent learning and memory including in mice [45]. We have previously shown that this is a sensitive test to detect learning and memory function recovery after neonatal hypoxia-ischemia induced brain injury [46]. However, we cannot exclude the possibility of long-term subtle changes in brain structure and functions in the present studies that were not detectable with the present methods, and this will need to be further investigated in the future.

Injury to the cerebellum is becoming increasingly recognized in preterm infants [47], [48]. Also in animal models, reduced number of neurons in cerebellum has been reported in the postnatal guinea-pig [49] and fetal sheep [50] following intrauterine growth-restriction. Moreover, a recent study found a diffuse pattern of cerebellar white matter damage in animals exposed to LPS while there was no obvious injury to the cerebellar cortex or of Purkinje cells [51]. In the present study, we found a significant decrease in the volume of the molecular layer after Pam_3_CSK_4_ treatment while there were no differences in the granule cell layer or number of Purkinje cells between groups. These observations suggest that TLR effects on the cerebellum may be region specific.

We found a significant increase in the number of microglia in Pam_3_CSK_4_ treated mice, but we saw no such increase in LPS treated animals. It is generally accepted that microglia are responsible for the innate immune response and that microglia express all TLRs, including TLR2 and TLR4, at readily detectable levels [52]. Thus, direct TLR2 stimulation could lead to the activation of microglia and release of pro-inflammatory cytokines, chemokines and free radicals, which could cause toxicity to neurons or oligodendrocytes [53], [54]. Indeed, levels of IL-1ß, IL-6, KC and MCP-1 significantly increased at 6 hours after the first Pam_3_CSK_4_ injection at PND3, indicating that the observed gray/white matter changes in the neonatal brain might be at least partly due to cytokine/chemokine toxicity to neurons/oligodendrocytes. Similar levels of most cytokines were seen after both Pam_3_CSK_4_ and LPS treatment, except for IL-1ß and IL-6, which was only significantly elevated following TLR2 agonist stimulation but not LPS stimulation. Of note, such observations may be consistent with the polarization of neonatal mononuclear cells towards relatively high TLR2-mediated IL-6 production [55]. Whether such differences in cytokine responses between Pam_3_CSK_4_ and LPS treatment contributed to the differences in microglia activation between these two treatments will be the subject of future investigation. Interestingly, IL-1ß is known to sensitize excitotoxic neonatal brain injury [56] and blocking of the IL-1ß receptor protects the immature brain from hypoxic-ischemic brain damage [57]. We did not observe differences in markers of proliferation or apoptosis at least not at PND 12 and 53, but decreased mature neuronal number suggests that effects of Pam_3_CSK_4_ on cell survival may have occurred at a time point prior to that examined.

The liver and spleen play a central role in immune responses and the liver is crucial in metabolizing microbial constituents such as Pam_3_CSK_4_. Thus the transient enlargement of the spleen and liver in Pam_3_CSK_4_ treated mice may indicate an acute reaction of the adaptive immune system and attempts to remove Pam_3_CSK_4_ in the blood.

Although our study demonstrates that repeated, high-dose, systemic administration of a TLR2 agonist can lead to CNS injury, it is important to note that these effects are likely context-dependent. Indeed, vaccines containing TLR2 agonists, including intradermal bacille Calmette-Guerin (BCG; *Mycobacterium bovis*) [58] and certain formulations of the intramuscular Haemophilus *influenzae* type b vaccine [59], have been safely and effectively administered to millions of infants. This underscores the importance of context, including route, frequency, and dose of administration when considering the impact of TLR agonists in injury models.

In conclusion, we found that systemic administration of a TLR2 agonist to neonatal mice caused transient gray and white matter injury in both the cerebrum and cerebellum. This suggests that engagement of the TLR2 pathway can have detrimental effects on the developing brain, and may play a role in neonatal brain injury associated with bacterial sepsis. However, neonatal brain injury is often multifactorial, and TLR2 agonist effects may interact with other exposures such as hypoxia/ischemia and/or be involved in a broader inflammatory response following Gram-positive bacterial exposure. Accordingly, it is possible that during Gram-positive bacterial infection, combined insults, including those driven via TLR2, may cause long-lasting functional or structural deficits.
